# The Trace Element Concentrations and Oxidative Stress Parameters in Afterbirths from Women with Multiple Pregnancies

**DOI:** 10.3390/biom13050797

**Published:** 2023-05-06

**Authors:** Konrad Grzeszczak, Patrycja Kapczuk, Patrycja Kupnicka, Donata Kinga Simińska, Joanna Lebdowicz-Knul, Sebastian Karol Kwiatkowski, Natalia Łanocha-Arendarczyk, Dariusz Chlubek, Danuta Izabela Kosik-Bogacka

**Affiliations:** 1 Department of Biology and Medical Parasitology, Pomeranian Medical University in Szczecin, Powstańców Wielkopolskich 72, 70-111 Szczecin, Poland; konrad.grzeszczak@pum.edu.pl (K.G.); natalia.lanocha.arendarczyk@pum.edu.pl (N.Ł.-A.); 2 Department of Biochemistry and Medical Chemistry, Pomeranian Medical University in Szczecin, Powstańców Wielkopolskich 72, 70-111 Szczecin, Poland; patrycja.kapczuk@pum.edu.pl (P.K.); patrycja.kupnicka@pum.edu.pl (P.K.); donata.siminska@pum.edu.pl (D.K.S.); dchlubek@pum.edu.pl (D.C.); 3 Department of Obstetrics and Gynecology, Pomeranian Medical University in Szczecin, Powstańców Wielkopolskich 72, 70-111 Szczecin, Poland; joanna.lebdowicz.knul@pum.edu.pl (J.L.-K.); sebastian.kwiatkowski@pum.edu.pl (S.K.K.); 4 Independent Laboratory of Pharmaceutical Botany, Pomeranian Medical University in Szczecin, Powstańców Wielkopolskich 72, 70-111 Szczecin, Poland

**Keywords:** iron, copper, zinc, antioxidant enzymes, lipid peroxidation, placenta, umbilical cord, fetal membrane, multiple pregnancies

## Abstract

The aim of this study was to evaluate the intensity of oxidative stress by measuring the concentrations of lipid peroxidation products (LPO) in fetal membrane, umbilical cord, and placenta samples obtained from women with multiple pregnancies. Additionally, the effectiveness of protection against oxidative stress was assessed by measuring the activity of antioxidant enzymes, including superoxide dismutase (SOD), catalase (CAT), glutathione peroxidase (GPX), and glutathione reductase (GR). Due to the role of iron (Fe), copper (Cu), and zinc (Zn) as cofactors for antioxidant enzymes, the concentrations of these elements were also analyzed in the studied afterbirths. The obtained data were compared with newborn parameters, selected environmental factors, and the health status of women during pregnancy to determine the relationship between oxidative stress and the health of women and their offspring during pregnancy. The study involved women (*n* = 22) with multiple pregnancies and their newborns (*n* = 45). The Fe, Zn, and Cu levels in the placenta, umbilical cord, and fetal membrane were determined using inductively coupled plasma atomic emission spectroscopy (ICP-OES) using an ICAP 7400 Duo system. Commercial assays were used to determine SOD, GPx, GR, CAT, and LPO activity levels. The determinations were made spectrophotometrically. The present study also investigated the relationships between trace element concentrations in fetal membrane, placenta, and umbilical cord samples and various maternal and infant parameters in women. Notably, a strong positive correlation was observed between Cu and Zn concentrations in the fetal membrane (*p* = 0.66) and between Zn and Fe concentrations in the placenta (*p* = 0.61). The fetal membrane Zn concentration exhibited a negative correlation with shoulder width (*p* = −0.35), while the placenta Cu concentration was positively correlated with placenta weight (*p* = 0.46) and shoulder width (*p* = 0.36). The umbilical cord Cu level was positively correlated with head circumference (*p* = 0.36) and birth weight (*p* = 0.35), while the placenta Fe concentration was positively correlated with placenta weight (*p* = 0.33). Furthermore, correlations were determined between the parameters of antioxidative stress (GPx, GR, CAT, SOD) and oxidative stress (LPO) and the parameters of infants and maternal characteristics. A negative correlation was observed between Fe and LPO product concentrations in the fetal membrane (*p* = −0.50) and placenta (*p* = −0.58), while the Cu concentration positively correlated with SOD activity in the umbilical cord (*p* = 0.55). Given that multiple pregnancies are associated with various complications, such as preterm birth, gestational hypertension, gestational diabetes, and placental and umbilical cord abnormalities, research in this area is crucial for preventing obstetric failures. Our results could serve as comparative data for future studies. However, we advise caution when interpreting our results, despite achieving statistical significance.

## 1. Introduction

Pregnancy is a period of rapid growth and cell differentiation. Both the mother and the fetus’ bodies are sensitive to changes in the supply of nutrients, including trace elements [1]. Therefore, it is important to ensure an optimal amount of these elements. Iron (Fe), zinc (Zn), and copper (Cu) are elements that serve as cofactors for many physiological processes related to the body’s homeostasis [2,3]. These elements also affect the development of organs and tissues as well as proper fetal weight gain [4,5,6], reducing the risks of preeclampsia [7,8,9] and preterm birth [10]. The role of Fe, Zn, and Cu during pregnancy, as well as the characteristics of the interactions between these elements, were described in a previous study by this team [11].

During pregnancy, women experience changes in their immune system that lead to the occurrence of inflammatory states characterized by the activation of macrophages and microphages, which produce high levels of reactive oxygen species (ROS) such as superoxide (O_2_^−•^), hydroxyl radicals (OH^−•^), and hydrogen peroxide (H_2_O_2_). The placenta is the main source of ROS during pregnancy [12], and the metabolism of ROS plays a crucial role in pregnancy progression, with increased levels of ROS observed in maternal blood [13]. However, the elevated ROS levels during pregnancy are counterbalanced by an increase in antioxidant synthesis [14]. An optimal level of ROS is necessary for many physiological processes, such as proliferation, defense reactions, signal transduction, and gene expression [15].

Oxidative stress (OS) arises due to an imbalance between ROS production and the body’s antioxidant defense mechanisms. OS in the placenta can lead to oxidative damage that can have far-reaching effects on the growth of the placenta and fetus. Complications during pregnancy such as miscarriage, preeclampsia, fetal growth restriction, and preterm birth have been linked to OS. Excessive ROS production can result in uncontrolled lipid peroxidation (LPO), which can cause disrupted membrane integrity and alterations in cellular metabolism. While LPO typically occurs at a low rate in all tissues and cells under normal physiological conditions, OS can lead to an increase in LPO.

Proteins such as superoxide dismutase (SOD), catalase (CAT), glutathione peroxidase (GPX), and glutathione reductase (GR) play a crucial role in protecting the body from oxidative stress. Of these, GPX and CAT are the main enzymes involved in neutralizing H_2_O_2_ [16]. The activity of SOD, CAT, and GPX has been observed to decrease in the placenta of women with preeclampsia [17,18,19], and lower CAT activity has been linked to premature births [20]. Optimal levels of SOD in the placenta protect the fetus from the adverse effects of oxygen by decreasing the concentration of lipid peroxides [21].

Trace elements, such as Fe, Zn, and Cu, also play a critical role in protecting against oxidative stress, with their availability being essential for enzymatic activity [22,23,24]. These elements can act as cofactors of enzymes involved in controlling free radicals in the body and are vital for antioxidant capacity [16]. Cu and Zn are structural components of transcription factors and SOD, and they act as signaling molecules [16]. Changes in Cu levels in the body can affect antioxidant systems and may play a role in pregnancy complications [16]. A deficiency of this micronutrient during pregnancy can lead to the development of oxidative stress, which may result in impaired fetal growth [25]. On the other hand, it has been shown that complications in the first trimester of pregnancy are more frequent in women with higher serum Cu levels than in those with lower levels. An inadequate Cu nutritional status leads to impaired antioxidant mechanisms, while an excessive Cu concentration promotes the production of reactive oxygen and nitrogen species [26]. A severe Zn deficiency in pregnant women can lead to fetal loss, fetal growth restriction, and low implantation rates [27]. Zn is a powerful antiradical and anti-inflammatory agent. Its ions form chelates with the sulfhydryl groups of proteins, shielding them from pro-oxidative processes. Zinc provides protection to cellular membranes from peroxidation by displacing Cu and Fe from their membrane-binding sites [16]. Zn is involved in the synthesis of antioxidant enzymes and acts as an enzymatic catalyst, playing a vital role in the metabolism of lipids, carbohydrates, and proteins. Along with Cu, Zn acts as a co-factor of Cu/Zn-SOD, which gets suppressed under Zn-deficient conditions [28]. Iron, like Cu, changes its oxidation state, leading to the production of reactive oxygen species, and is a component of SOD. Both Fe deficiency and excess during pregnancy are detrimental to fetal development [29]. The research has indicated that excess Fe saturation during the prenatal period may elevate the risks of miscarriage, premature birth, low birth weight, and being small for gestational age (SGA), with OS being one of the likely mechanisms behind these abnormalities. In pregnant women without Fe deficiency anemia (IDA), prophylactic Fe supplementation has been observed to trigger oxidative stress and limit the organism’s antioxidant capacity [30,31].

Limited data are available on the parameters of OS and antioxidant defense in afterbirths from women with multiple pregnancies [32], who have higher risks of mortality and morbidity [33]. Multiple pregnancies are associated with abnormalities in the placenta, which can lead to intrauterine growth restriction and abnormalities in the umbilical cord [34]. Therefore, the objective of this study was to assess the intensity of oxidative stress by quantifying the concentrations of lipid peroxidation products in fetal membrane, umbilical cord, and placenta samples collected from women with multiple pregnancies. Additionally, the effectiveness of protection against OS was evaluated by measuring the activity of antioxidant enzymes, namely SOD, CAT, GPX, and GR. In view of the role of Fe, Cu, and Zn as cofactors for antioxidant enzymes, the concentrations of these elements were also analyzed in the studied afterbirths. The obtained data were compared with the newborn parameters, selected environmental parameters, and maternal health during pregnancy.

## 2. Materials and Methods

### 2.1. Ethics Statement

The study was conducted between 2015 and 2021, following the approval of the Biometric Committee of the Pomeranian Medical University in Szczecin (KB-0012/76/14 from 13 October 2014). The patients provided written informed consent to participate and were informed of their right to withdraw their consent at any point during the study. The research was conducted in accordance with the principles of the Declaration of Helsinki.

### 2.2. Study Population

The study enrolled 22 European women with multiple pregnancies and their newborns (*n* = 45) at the Obstetrics and Gynecology Clinic of the Independent Public Clinical Hospital No. 2 of the Pomeranian Medical University in Szczecin, northwest Poland. The multiple pregnancies included 21 twin pregnancies, including diamniotic (*n* = 14), monochorionic diamniotic (*n* = 5), and monochorionic monoamniotic (*n* = 2) twin pregnancies, and 1 triple pregnancy (dichorionic triamniotic triplets), all of which were terminated by cesarean section. The study’s inclusion criteria were multiple pregnancies and newborns without perinatal illness, and the sample was randomly selected. All women were healthy and had no risk factors that could affect the neonatal parameters, with those having hypertension and diabetes excluded. Infants with anemia, chromosomal abnormalities, or birth defects were also excluded. Table 1 and Table 2 present the newborns’ characteristics.

At birth, standard anthropometric procedures were used to measure the birth weight, length, and head circumference. The gestational age was determined by the crown–rump length measured in the first trimester using ultrasonography and calculated based on the first day of the last menstrual period using Naegele’s rule. The mothers’ anthropometric and biological characteristics, including their age, weight, and a morphological blood analysis, as well as the infants’ shoulder width, weight, length, head circumference, gestational age, and sex, were obtained from medical records. Additionally, data on the weight of the placenta and length of the umbilical cord were also collected from medical records.

The socio-demographic information, smoking history prior to pregnancy (*n* = 6), and obstetric and gynecological histories, including parity (number of previous deliveries), were collected through general questionnaires. During periodic medical interviews, the majority of women (*n* = 19) reported taking Prenatal DUO©, a daily iron (II) fumarate supplement with a 30 mg dose. The body mass index (BMI) was calculated according to a women’s weight in kilograms divided by the square of height in meters [35]; only 18 women were in the range of 18.5–24.9. Unfortunately, data regarding maternal dietary habits during pregnancy were not available, although there were no indications of nutritional impairment or malnutrition among the participants.

Immediately following delivery, placentas (*n* = 38), umbilical cords (*n* = 45), and fetal membranes (*n* = 42) were collected, weighed, measured, and stored at −80 °C until the study group had been assembled.

### 2.3. Determination of Metals in Afterbirths

The samples were first restored to room temperature and then dried for three weeks at 105 °C to determine their water content (gravimetric method), following the protocols of Kalisinska et al. [36] and Lanocha et al. [37]. The dried samples were then ground into a fine powder using a porcelain mortar and subsequently mineralized [38]. The levels of Fe, Cu, and Zn were analyzed through inductively coupled plasma atomic emission spectroscopy (ICP-OES) using an ICAP 7400 Duo instrument from Thermo Scientific (Waltham, MA, USA), with the wavelengths (nm) of Fe = 238.204, Zn *=* 213.856, and Cu = 224.700 being utilized. In order to validate the analytical techniques, samples of Bovine Muscle NIST-SRM 8414 reference material (National Institute of Standards and Technology, Gaithersburg, MD, USA) were also assayed. The concentrations of metals in the reference material provided by the manufacturers and our own determinations are shown in Table 3. The Fe, Cu, and Zn concentrations in the placenta, umbilical cord, and fetal membrane are presented in mg/kg^−1^ dry weight (dw).

### 2.4. Determination of Oxidative Stress in Afterbirths

The tissue fragments were ground with a mortar and pestle, and pulverized and frozen samples were placed into a tube containing 500 µL of an appropriate buffer, following the protocol of a commercial enzyme assay kit, while being maintained at 4 °C. The material was vortexed and homogenized using a knife homogenizer. The resulting extract mixtures were centrifuged (3000× *g* for 10 min, at 4 °C) to obtain the supernatant, which was used for enzyme quantification.

The following reagent kits were used to determine the antioxidant enzyme activity: a Superoxide Dismutase Assay Kit (Cayman Chemical, Ann Arbor, MI, USA), Catalase Assay Kit (Cayman Chemical, Ann Arbor, MI, USA), Glutathione Peroxidase Assay Kit (Cayman Chemical, Ann Arbor, MI, USA), Glutathione Reductase Assay Kit (Cayman Chemical, Ann Arbor, MI, USA), and Lipid Peroxidation (LPO) Assay Kit (G-bioscience, St Louis, MO, USA). The determinations were made spectrophotometrically in accordance with the protocols provided by the manufacturers and are presented in U/mg protein^−1^.

### 2.5. Statistical Analysis

A statistical analysis was performed using Statistica v13.0 (Stat Soft). A Shapiro–Wilk analysis was performed to test the normal distribution of the data. Due to the non-normal distribution of data, the Spearman rank correlation was used to analyze the relationship between the variables, determining the value of the coefficient and the level of statistical significance (rho, ρ). In order to assess differences between the parameters, a Kruskal–Wallis ANOVA followed by Mann–Whitney-U tests were used. The significance level was *p* < 0.05.

## 3. Results

The mean water constituents in the placenta, fetal membrane, and umbilical cord equaled approximately 83%, 85%, and 88%, respectively. In the scientific literature, metal concentrations are given in terms of the dry (mg kg^−1^ dw) or wet weight (mg kg^−1^ ww) of tissues and organs. In order to make it possible to compare our own results with the literature data expressed in mg kg^−1^ ww, aconversion factor of 1.5 was used in this study, since on average the afterbirths contained at least 85% water.

**Figure 1:**
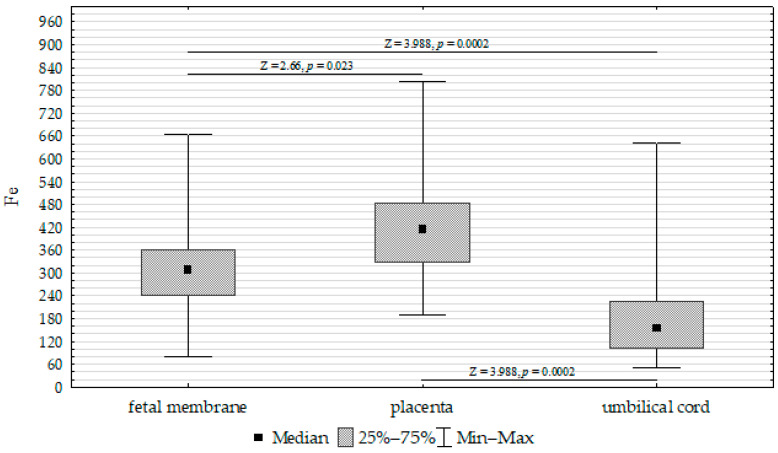
Kruskal–Wallis test results for differences in iron (Fe) concentrations between the placenta, fetal membrane, and umbilical cord.

**Figure 2:**
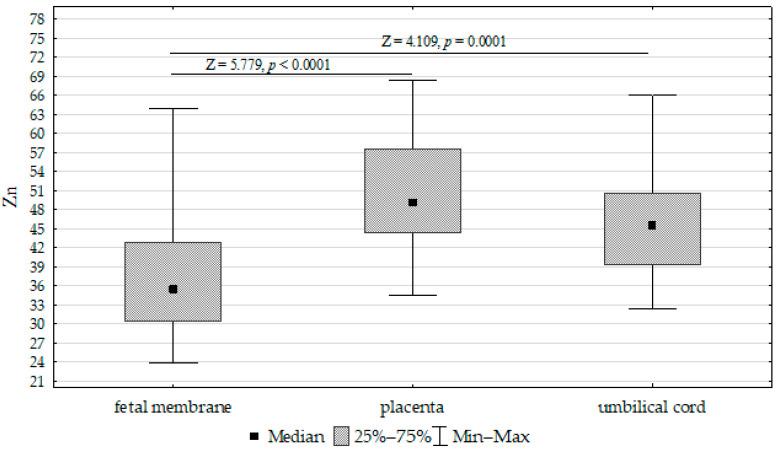
Kruskal–Wallis test results for differences between zinc (Zn) concentrations in the placenta, fetal membrane, and umbilical cord.

**Figure 3:**
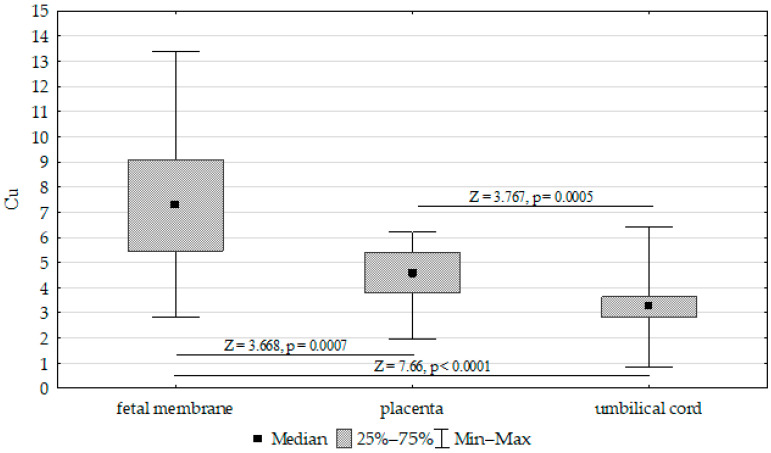
Kruskal–Wallis test results for differences in cooper (Cu) concentrations between the placenta, fetal membrane, and umbilical cord.

The concentrations of Fe, Zn, and Cu in the placenta, umbilical cord, and fetal membrane are presented in Table 4. The highest concentration of Fe (417.52 mg/kg^−1^ dw) was found in the placenta, while the lowest was found in the umbilical cord (200.17 mg/kg^−1^ dw). The zinc concentration was highest in the placenta (50.21 mg/kg^−1^ dw) and lowest in the fetal membrane (36.91 mg/kg^−1^ dw). In contrast, the highest concentration of Cu was found in the fetal membrane (7.31 mg/kg^−1^ dw), while the lowest was found in the placenta (4.47 mg/kg^−1^ dw). Accordingly, the Fe, Cu, and Zn concentrations differed significantly between the umbilical cord, placenta, and fetal membrane (Figure 1, Figure 2 and Figure 3).

In the study, we determined correlations between Fe, Cu, and Zn concentrations in the placenta, umbilical cord, and fetal membrane and the parameters of the infants, maternal characteristics, and gestational age. In the studied tissues from women of Poland, we found strong positive correlations between Cu and Zn concentrations in the fetal membrane (*p* = 0.66) and Zn and Fe concentrations in the placenta (*p* = 0.61). We found a correlation between the fetal membrane Zn concentration and shoulder width (*p* = −0.35); between the placenta Cu concentration and (i) placenta weight (*p* = 0.46) and (ii) shoulder width (*p* = 0.36); between the umbilical cord Cu level and (i) head circumference (*p* = 0.36) and (ii) birth weight (*p* = 0.35); and between the placenta Fe concentration and placenta weight (*p* = 0.33).

**Figure 4:**
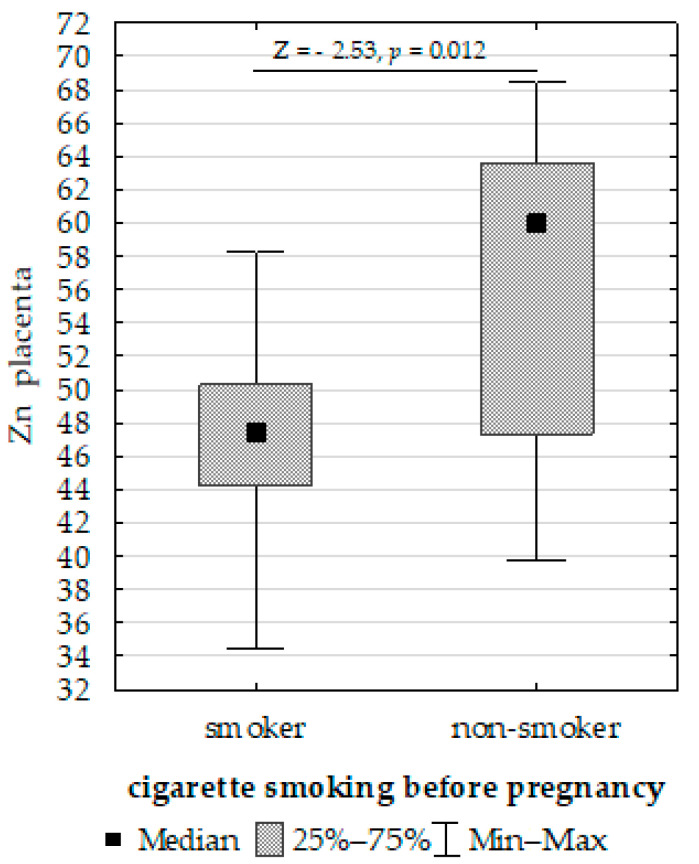
Mann–Whitney U test results for differences in placenta zinc (Zn) between non-smoking women and those smoking before pregnancy.

**Figure 5:**
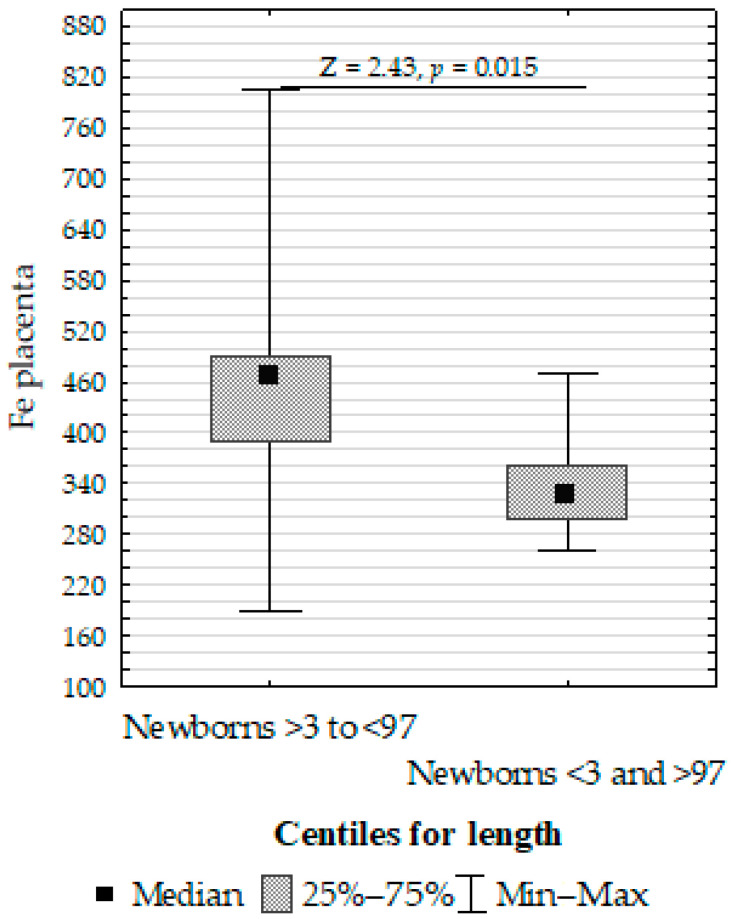
Mann–Whitney U test results for the differences in placenta iron (Fe) between the >3 to <97 centiles and <3 and >97 centiles groups.

**Figure 6:**
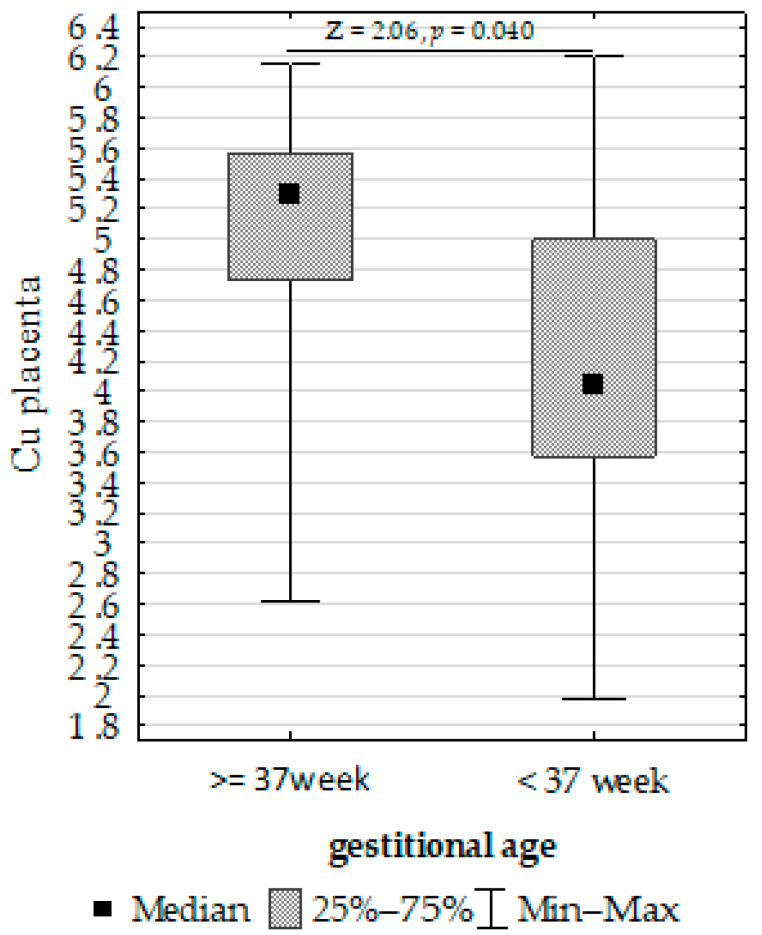
Mann–Whitney U test results for differences in placenta copper (Cu) depending on gestational age.

Furthermore, we compared the Fe, Cu, and Zn concentrations in the placenta, umbilical cord, and fetal membrane and the parameters of the infants, gestational age, maternal characteristics, supplementation, and cigarette smoking before pregnancy. The Mann–Whitney U test showed the following:

- Upregulation of placenta Zn in non-smoking women compared to those smoking before pregnancy (Figure 4);
- Upregulation of placenta Fe in newborns with >3 or <97 centiles for length compared to <3 or >97 (Figure 5);
- Upregulation of placenta Cu at ≥37 weeks of gestation compared to <37 weeks of gestation (Figure 6).

In addition, the concentrations of LPO and the activities of antioxidant enzymes (SOD, CAT, GPX, GR) were analyzed in fetal membranes, umbilical cords, and placentas obtained from women with multiple pregnancies, and the results are presented in Table 5. In the studied afterbirths, the activity of antioxidant enzymes (as measured by the AM assay) was observed to decrease in the following order:

- For GPx: fetal membrane > placenta > umbilical cord;
- For GR: placenta > umbilical cord > fetal membrane;
- For CAT and SOD: umbilical cord > fetal membrane > placenta.

**Table 5:** The concentrations of antioxidants and pro-oxidants in the placenta, umbilical cord, and fetal membrane (U/mg protein^−1^) (AM, arithmetic mean; Med, median; Max, maximum; Min, minimum; SD, standard deviation; GPx, glutathione peroxidase; GR, glutathione reductase; CAT, catalase; SOD, superoxide dismutase; LPO, lipid peroxidation product levels).

	Placenta			Umbilical Cord			Fetal Membrane		
	AM ± SD	Med.	Range	AM ± SD	Med.	Range	AM ± SD	Med	Range
Total (*n* = 22) = 21 twins + 1 triplets Parameters antioxidative stress									
SOD	0.04 ± 0.03	0.04	0.01–0.14	0.09 ± 0.07	0.07	0.01–0.29	0.06 ± 0.07	0.04	0.01–0.30
CAT	1 ± 1	0.69	0.17–8.26	3 ± 2	2.90	0.76–8.34	2 ± 2	1.46	0.27–8.83
GPx	5 ± 6	3.14	0.10–36.07	4 ± 4	3.82	0.30–18.31	6 ± 6	4.49	0.33–25.64
GR	6 ± 3	5.24	0.56–14.26	4 ± 3	3.52	0.14–13.24	4 ± 3	3.40	0.18–16.98
SOD/GPx ratio	0.03 ± 0.07	0.01	0.01–0.39	0.04 ± 0.05	0.02	0.01–0.26	0.02 ± 0.02	0.01	0.01–0.11
SOD/CAT ratio	0.07 ± 0.08	0.04	0.01–0.31	0.06 ± 0.17	0.03	0.01–1.1	0.03 ± 0.02	0.03	0.01–0.11
Parameters oxidative stress									
LPO	2 ± 1	2.26	0.04–4.19	2 ± 1	1.41	0.04–6.09	2 ± 1	1.42	0.11–4.67

The highest concentration of LPO was found in the placenta, while lower concentrations were observed in the umbilical cord and fetal membrane.

To assess whether the antioxidant defense mechanism is sufficient in the afterbirths, we investigated the ratios of SOD/GPx and SOD/CAT (Table 5). The highest SOD/GPx ratio was observed in the umbilical cord, while the SOD/CAT ratios were similar in all of the investigated tissues. We observed significant differences in SOD/GPx and SOD/CAT ratios between some of the parameters analyzed, as follows: SOD/GPx and GPx in the fetal membrane (*p* = −0.67); SOD/GPx and SOD in the fetal membrane (*p* = 0.64); SOD/GPx and GPx in the placenta (*p* = −0.85); SOD/GPx and SOD in the placenta (*p* = 0.39); the placenta SOD/GPx and Cu in the umbilical cord (*p* = 0.35); SOD/GPx and GPx in the umbilical cord (*p* = −0.55); SOD/GPx and SOD in the umbilical cord (*p* = 0.60); the fetal membrane SOD/CAT and GPx in the umbilical cord (*p* = 0.32); SOD/CAT and CAT in the fetal membrane (*p* = −0.45); SOD/CAT and SOD in the fetal membrane (*p* = 0.70); SOD/CAT and GR in the placenta (*p* = −0.37); SOD/CAT and CAT in the placenta (*p* = −0.70); SOD/CAT and SOD in the placenta (*p* = 0.41); placenta SOD/CAT and SOD in the umbilical cord (*p* = −0.55); the umbilical cord SOD/CAT and placenta GPx (*p* = −0.36); the umbilical cord SOD/CAT and placenta CAT (*p* = 0.47); SOD/CAT and SOD in the umbilical cord (*p* = 0.82); SOD/CAT and Zn in the umbilical cord (*p* = 0.36); SOD/CAT and Cu in the umbilical cord (*p* = 0.52).

Additionally, we determined correlations between parameters of antioxidative stress (GPx, GR, CAT, SOD) and oxidative stress (LPO) and the parameters of the infants and maternal characteristics (Table 6). We found negative correlations between Fe and LPO products concentrations in the fetal membrane (*p* = −0.50) and placenta (*p* = −0.58) and the Cu concentration and SOD activity in the umbilical cord (*p* = 0.55) (Table 6).

Furthermore, we determined the correlations between antioxidative defense (SOD, CAT, GPx, GR) and oxidative stress (LPO) in the placenta, umbilical cord, and fetal membrane, and the characteristics of the infants and BMI (Table 6). 

**Figure 7:**
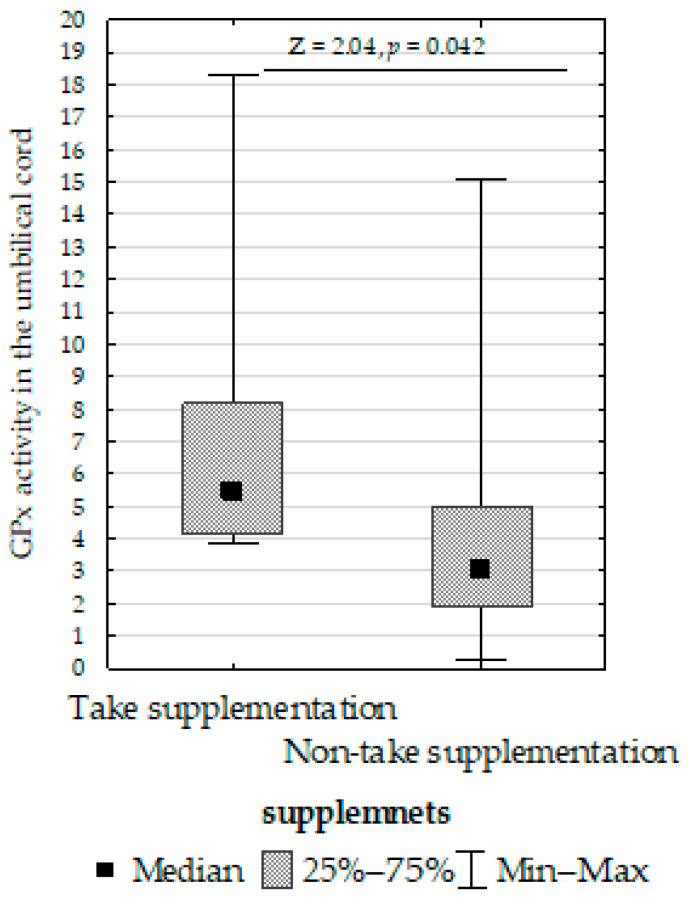
Mann–Whitney U test results for differences in glutathione peroxidase (GPx) activity between women taking supplementation and those not taking supplementation.

**Figure 8:**
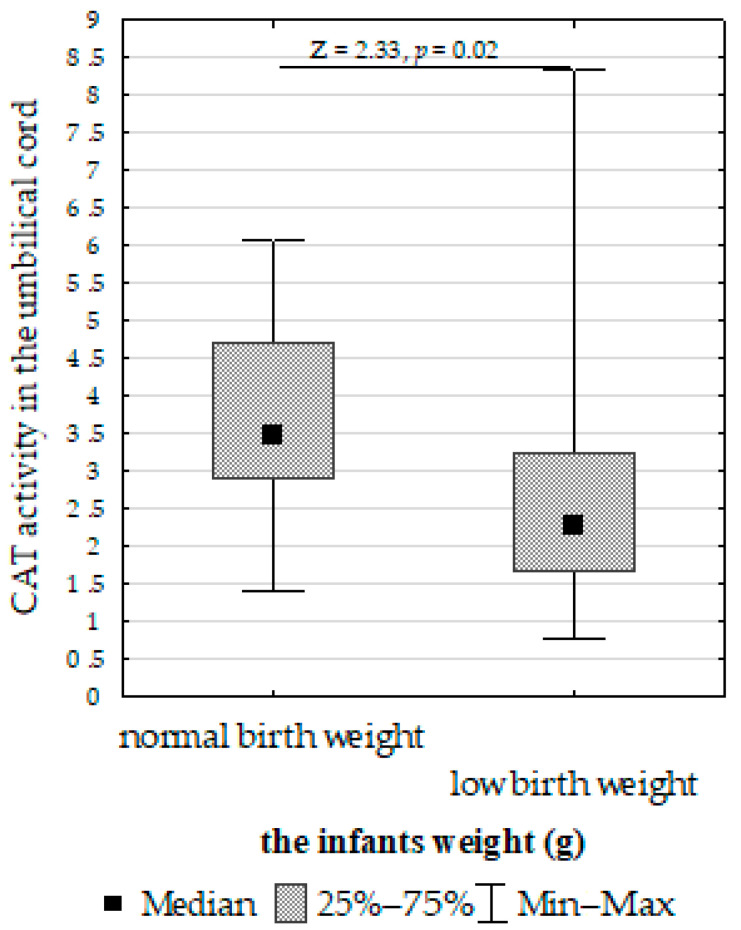
Mann–Whitney U test results for differences in catalase (CAT) activity between infants with normal and low birth weights.

**Figure 9:**
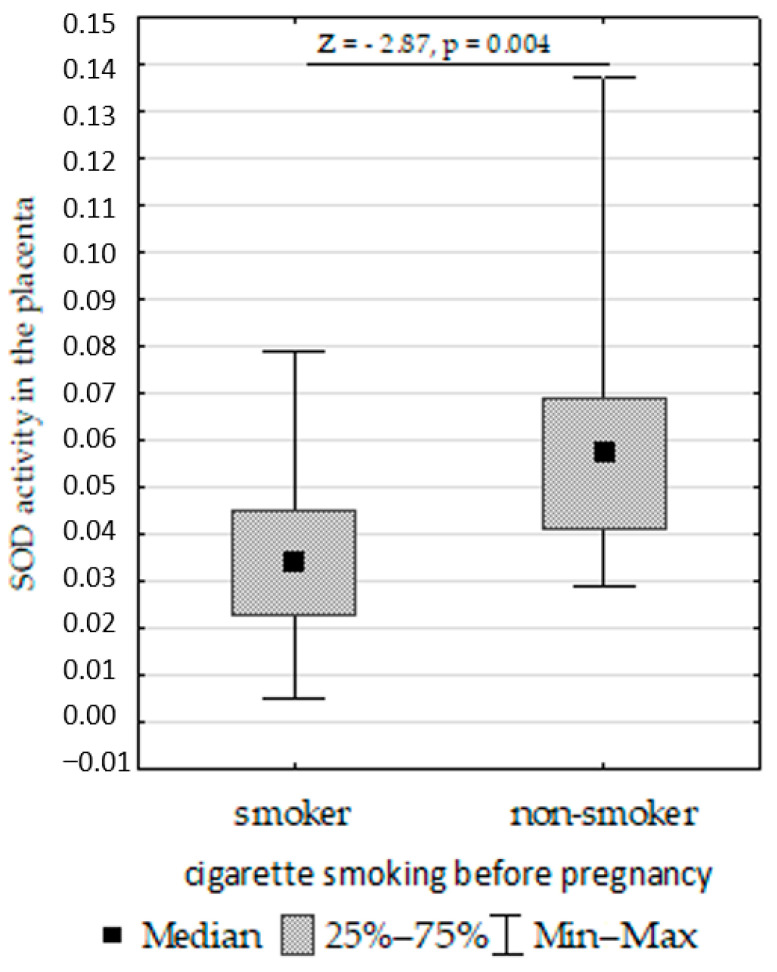
Mann–Whitney U test results for differences in superoxide dismutase (SOD).

**Figure 10:**
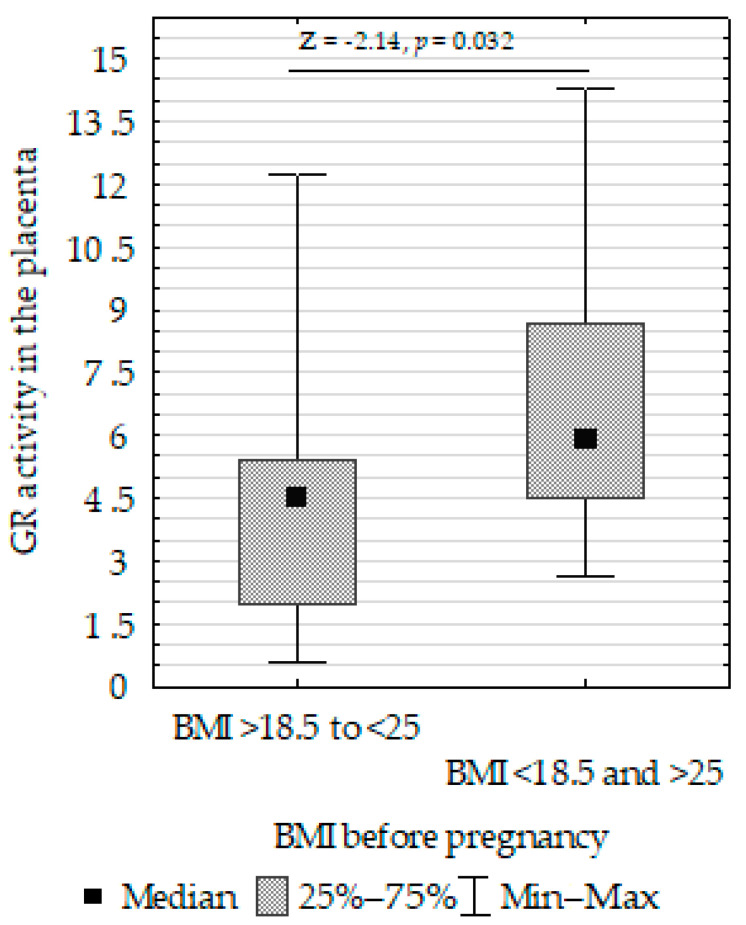
Mann–Whitney U test results for the differences in placenta GR activity between non-smoking women and those smoking before pregnancy activity between the >18.5 to <25 BMI and <18.5 and >25 BMI groups.

We compared SOD, CAT, GPx, GR, and LPO in the placenta, umbilical cord, and fetal membrane and the parameters of the infants, gestational age, maternal characteristics, supplementation, and cigarette smoking before pregnancy. The Mann–Whitney U test showed the following:

- Decreased umbilical cord GPx activity in women who did not take supplementation compared to those who did (Figure 7);
- Increased umbilical cord CAT activity in infants with normal birth weight compared to those with low birth weight (Figure 8);
- Increased placenta SOD activity concentration in non-smoking women compared to those who smoked before pregnancy (Figure 9);
- Decreased placenta GR activity in women with >18.5 to <25 BMI compared to women with <18.5 and >25 BMI (Figure 10).

## 4. Discussion

### 4.1. Trace Element Concentrations in Afterbirth

Essential trace elements such as Fe, Cu, and Zn play a critical role in the normal progression of pregnancy and act as cofactors for antioxidant enzymes involved in enzymatic defense mechanisms against oxidative stress. However, there is currently a lack of research on the levels of these elements in the placenta, umbilical cord, and fetal membrane of women with multiple pregnancies. In this study, the concentrations of these elements were compared to those found in afterbirths obtained from women with singleton pregnancies.

### 4.2. Iron Concentration in Afterbirth

In this study, the mean Fe concentrations were found to be 417.5 mg/kg^−1^ dw in the placenta, 200.17 mg/kg^−1^ dw in the umbilical cord, and 317.10 mg/kg^−1^ dw in the fetal membrane as obtained from women with multiple pregnancies. Comparing these results with our previous research on Fe concentrations in afterbirths obtained from women with singleton pregnancies from the same study area, higher Fe concentrations were found in the placenta (640.73 mg/kg^−1^ dw), umbilical cord (640.73 mg/kg^−1^ dw), and fetal membrane (567.29 mg/kg^−1^ dw) [39]. However, Barad et al. [40] reported lower Fe concentrations in the placenta obtained from teenage women with singleton pregnancies (71.1 mg/kg^−1^ ww; 106.7 mg/kg^−1^ dw after conversion) than in women aged 20–46 years with multiple pregnancies (84.6 and 78.6 mg/kg^−1^ ww; 126.9 and 117.9 mg/kg^−1^ dw after conversion, respectively, for twin and triplet placentas). De Angelis et al. [41] reported that the Fe concentration in the placenta was higher in women with multiple pregnancies (26.05 μg/g^−1^ dw) than in those with singleton pregnancies (17.99 μg/g^−1^ dw), although this difference was not statistically significant. These differences may have been due to differences between the study groups.

Mbofung et al. [42] demonstrated higher Fe concentration in the placenta of female newborns than in those of male newborns. Reddy et al. [43] reported that the Fe concentration in the placenta of women from urban areas was higher than that of women from rural areas. Additionally, Irwinda et al. [44] found that the Fe concentration in the placenta of premature newborns was lower than that of full-term newborns.

In the present study, it was found that the concentration of Fe in the placenta had an impact on its weight, as confirmed by the analyses of Hindmarsh et al. [45] in women with singleton pregnancies. Godfrey et al. [46] demonstrated that Fe deficiency is associated with a larger placental weight, which may have significant implications for the infant. Levario-Carrillo et al. [47] confirmed a tendency for increased placental weight in women with iron deficiency anemia (IDA); however, there was no significant effect on the weight or growth of the newborn. On the other hand, other studies have shown that IDA during pregnancy can lead to low birth weight [48,49,50].

In the present study, no association was found between Fe supplementation and neonatal morphometric parameters. However, Shi et al. [51], in their study of women and newborns from singleton pregnancies, reported that Fe supplementation during pregnancy had an effect on increasing the birth weight of newborns, particularly in women with anemia. However, it is important that the supplementation is individually tailored, as the excessive intake of Fe may have negative effects on fetal development. Hwang et al. [52] demonstrated that increased maternal Fe intake can lead to fetal growth restriction. On the other hand, Preziosi et al. [53] found that the mean newborn length and Apgar score were higher in the group of pregnant women receiving Fe-containing supplements than in the group receiving a placebo. The relationship between Fe supplementation and higher birth weight has been confirmed, indicating that Fe supplementation may have an impact on neonatal morphometric parameters [54,55].

### 4.3. Zinc Concentration in Afterbirth

In the present study, the average concentrations of Zn in the placenta, umbilical cord, and fetal membrane obtained from women with multiple pregnancies from northwestern Poland were 50.21, 45.55, and 36.91 mg/kg^−1^ dw, respectively. Much higher concentrations of Zn in the placenta (66.90 mg/kg^−1^ dw), umbilical cord (54.65 mg/kg^−1^ dw), and fetal membrane (62.79 mg/kg^−1^ dw) were found in women with singleton pregnancies from the same study area [39]. De Angelis et al. [41] found that the Zn concentration in the placenta was higher in women with multiple pregnancies (5.9 μg/g^−1^ dw) than in those with singleton pregnancies (2.6 μg/g^−1^ dw), but the difference was not statistically significant. Mazurek et al. [56] reported much lower Zn concentrations in the placenta (ranging from 3.3 to 11.5 mg/kg^−1^ ww; ranging from 5.0 to 17.3 mg/kg^−1^ dw after conversion) obtained from women with singleton pregnancies in Poland. Kantola et al. [57] found higher concentrations of Zn in the placenta obtained from non-smoking women with singleton pregnancies from Finland, Estonia, and St. Petersburg (77.14 mg/kg^−1^ dw) than in the present study.

The present study showed differences in the placental concentrations of Zn between women who smoked and did not smoke before pregnancy. Kantola et al. [47] observed that the Zn concentration in the placenta of women who smoked during pregnancy was 20% higher than that of non-smoking women in a study involving women with singleton pregnancies from various regions in Europe. In contrast, Kutlu et al. [58] found that the concentration of Zn in the placenta of smoking women was lower than that of non-smoking women during pregnancy. These results suggest that changes in Zn concentration caused by nicotine may be due to the vasoconstriction of utero placental blood vessels, thereby disrupting the transport of nutrients, including macro- and micronutrients. Additionally, nicotine may contribute to the destruction of the epithelial fibers of the placenta responsible for the transport of these elements [59].

It has been observed that primary Zn deficiency may lead to fetal growth retardation, confirming the important role of Zn in normal prenatal child development [60]. Bermúdez et al. [61] found no association between anthropometric parameters and Zn levels in either umbilical or maternal blood. Kot et al. [62] found a negative correlation between the maternal blood Zn levels and newborn chest circumference, as well as between umbilical blood Zn levels and newborn head circumference. In the present study, a correlation was found only between the Zn concentration in the fetal membrane and the child’s shoulder width. A cohort study showed that Zn deficiency in pregnant women increases the risk of low birth weight (LBW) in newborns [63]. Simmer and Thompson [64] observed a relationship between a low maternal plasma Zn concentration and decreased small-for-gestational-age newborn ratio, while Lira et al. [65] demonstrated that Zn supplementation slightly increased birth weight. Sorouri et al. [66] found no significant impact on Zn sulfate daily supplementation (15 mg) from the 16th week of gestation until delivery in a randomized controlled trial.

### 4.4. Cooper Concentration in Afterbirth

In the present study, the mean Cu concentrations in the placenta, umbilical cord, and fetal membrane obtained from women with multiple pregnancies from northwestern Poland were 4.47, 3.21, and 7.26 mg/kg^−1^ dw, respectively. Much higher concentrations of Cu in the placenta (6.01 mg/kg^−1^ dw), umbilical cord (4.32 mg/kg^−1^ dw), and fetal membrane (8.91 mg/kg^−1^ dw) were found in women with singleton pregnancies from the same study area [39]. Much lower concentrations of Cu in the placenta (from 0.05 to 3.91 μg/g^−1^ ww; from 0.08 to 5.9 ug/g^−1^ dw after conversion) were reported by Mazurek et al. [56]. Higher concentrations of Cu in the placenta were found by Reddy et al. [43] in pregnant women from rural (78.4 μg/g^−1^ dw) and urban (61.4 μg/g^−1^ dw) areas, as well as in term newborns and preterm infants [44].

In addition, in the presented study, it was found that the concentration of Cu in the placenta significantly correlates with the placenta weight and shoulder width, and the concentration of Cu in the umbilical cord correlates with the head circumference. Kot et al. [39] showed a negative correlation between the concentration of Cu in fetal membranes collected from women with singleton pregnancies and the birth weight of newborns. Furthermore, Kot et al. [39] found a negative correlation between the concentration of Cu in umbilical cord blood collected from women with singleton pregnancies and newborn parameters (head circumference, chest circumference, and Apgar score in the first minute). Mbofung and Subbarau [42] demonstrated a positive correlation between the concentration of Cu in the placenta collected from women with singleton pregnancies and newborn birth weight. Kantola et al. [57] found a negative correlation between the Cu concentration in the placenta and the newborn birth weight, which was stronger in the group of smoking women than non-smoking women.

### 4.5. The Relationships between Essential Elements

The metabolism of Fe and Cu is interrelated, and during pregnancy, both a deficiency of Cu and an excess of Fe can affect each other [67]. In women, it has been found that Fe and Cu may competitively interact with each other at the stage of intestinal absorption, and Fe supplementation may decrease Cu levels in the body [68]. It has been observed that in cases of Fe deficiency in the placenta, the level of Cu increases to a higher level than in other tissues [69].

In the present study, a correlation was observed between the Fe concentration in the placenta and Zn and Cu concentrations in the placenta, and between the Fe level in the fetal membrane and Zn concentration in the fetal membrane. Additionally, a positive correlation was found between the Zn and Fe concentrations in the placenta and between the Zn concentration in the placenta and Cu levels in the placenta and umbilical cord, as well as between the Zn concentrations in the fetal membrane and umbilical cord and the Cu concentrations in these structures. Kinnamon [70] noted that Cu and Zn in the diet induce a competitive mechanism within the fetal and placental structures. Kantola et al. [57] observed a negative correlation between the Cu concentration in the placenta and the birth weight of the newborn.

Deficiencies of both Zn and Cu are well described in preterm infants and pregnant women [71], but not in the case of structures such as the placenta, fetal membrane, and umbilical cord from women with multiple pregnancies. It can be inferred that the antagonistic effects of Zn and Cu, Zn and Fe, and Cu and Fe may also occur at the level of maternal–fetal and fetal–placental, fetal membrane, and umbilical cord interactions.

### 4.6. Oxidative Stress and Fe, Cu, and Zn Concentrations

The human body naturally produces free radicals [72], but their uncontrolled production can cause lipid peroxidation and damage to cell membranes [73]. To prevent excessive OS, the body uses enzymatic and non-enzymatic antioxidants to scavenge free radicals and limit their concentrations [74].

During pregnancy, increases in lipid peroxidation and oxidative stress may pose a potential threat to the developing fetus. To counteract these adverse effects, changes are made to the fetal ROS defense system. However, this adaptive mechanism may not always provide adequate protection against harm [75]. Multiple pregnancies entail an even greater risk of oxidative stress and reduced antioxidant capacity, potentially linking oxidative stress to pregnancy complications [76]. For instance, Minghetti et al. [32] noted a correlation between oxidative stress and lower birth weight in newborns.

In the present study, the mean concentrations of GPx in the placenta, umbilical cord, and fetal membrane were 5.19, 4.45, and 6.37 U/mg protein^−1^, respectively. Mistry et al. [77] found that the GPx activity was lower in the placental tissues of women with preeclampsia than in normotensive women. Similarly, Biri et al. [78] observed that the GPx activity was lower in pregnant women with preeclampsia than in healthy women.

The mean concentrations of GR in the placenta, umbilical cord, and fetal membrane were 5.55, 4.05, and 3.85 U/mg protein^−1^, respectively. Das et al. [79] did not find significant differences in GR activity in the placentas of women with preeclampsia and healthy pregnant women.

The mean concentrations of CAT in the placenta, umbilical cord, and fetal membrane were 1.17, 3.21, and 2.28 U/mg protein^−1^, respectively. Ferreira et al. [80] found that the CAT activity was lower in women with preeclampsia than in healthy pregnant women.

The SOD activity levels in the placenta, umbilical cord, and fetal membrane were 0.04, 0.09, and 0.06 U/mg protein^−1^, respectively. Biri et al. [78] reported that the SOD activity in pregnant women with preeclampsia was similar to that of healthy pregnant women. Ferreira et al. [80] found that the SOD activity was higher in women with preeclampsia than in healthy pregnant women.

In the present study, it was observed that LPO was highest in the placenta (2.26 U/mg protein^−1^), followed by the umbilical cord (1.66 U/mg protein^−1^) and fetal membrane (1.65 U/mg protein^−1^). Similarly, Ohel et al. [81] reported that LPO was significantly higher in fetal membranes compared to the placenta. Furthermore, the study revealed that the concentrations of Fe in the placenta and fetal membrane affect the level of oxidative stress by modulating the antioxidant parameters in the placenta (GPx, GR) and umbilical cord (GPx).

Several researchers have confirmed the positive effect of Fe on antioxidant defense parameters [82,83,84]. Bozkaya et al. [85] demonstrated that enzymatic antioxidant markers were reduced in women with iron-deficiency anemia (IDA). The deficiency of Fe in women with IDA may intensify OS, causing changes in the function of the heart. Isler et al. [86] found that Fe had a positive effect on the activity of SOD and GPx. Researchers have noted that the activity of SOD is lower in patients with IDA and that Fe supplementation in patients with IDA restores optimal antioxidant activity. Excess Fe predisposes individuals to increased LPO activity [87,88]. Lachili et al. [30] demonstrated that pharmacological doses of Fe administered to pregnant women without anemia caused uncontrolled lipid peroxidation. Similarly, Rajendran et al. [89] found that Fe supplementation in non-anemic pregnant women resulted in increased oxidative stress and inflammation. The present study confirmed the relationship between the concentration of Fe in the placenta samples obtained from women with multiple pregnancies and LPO activity.

Metalloproteins (CAT and SOD) act as antioxidants by enzymatically detoxifying reactive oxygen species, including O_2_, H_2_O_2_, and -OOH. Cu/Zn superoxide dismutase, which is especially demanding for its proper function and dependent on its cofactors (Cu/Zn), was observed to be correlated with placental Cu and Zn concentrations and umbilical cord Cu and Zn levels in the present study. Additionally, a correlation was observed between the fetal membrane Cu level and umbilical cord Cu concentration with the fetal membrane CAT and umbilical cord Cu level with placenta CAT. The maintenance of pregnancy is significantly influenced by appropriate Cu/Zn SOD activity, according to several studies [90,91,92]. Cu/Zn SOD activity has been found to decrease in the placenta of women with gestational diabetes [93] and preeclampsia [94], indicating a possible link between Cu/Zn SOD activity and pregnancy complications. However, conflicting findings have been reported by Araújo Brito et al. [95], who suggest that SOD may not be a useful marker for preeclampsia, as the activity significantly increased in both groups (women with and without preeclampsia).

Oxidative stress can affect fetal anthropometric parameters, particularly birth weight [96,97]. In this study, SOD activity in the placenta was found to correlate with the birth weight and head circumference. Additionally, SOD activity in the umbilical cord correlated with the birth weight, length, and shoulder width. Saker et al. [98] demonstrated increased SOD activity in cases of intrauterine growth restriction (IUGR). Similarly, Biri et al. [78] found increased SOD activity in newborns with IUGR and suggested that the administration of antioxidants could be useful in the prevention or treatment of IUGR.

CAT activity affects newborn morphometric parameters [99]. In the present study, higher CAT activity was found in the group of newborns with normal weight compared to the group with low weight. Additionally, fetal membrane CAT activity correlated with head circumference and birth weight. Ordóñez-Díaz et al. [100] associated extra uterine growth restriction with an impaired antioxidant defense status. Similarly, Karowicz-Bilinska et al. [101] and Luo et al. [102] demonstrated that IUGR is associated with greater placental ROS and oxidative injury. Ashin et al. [96] noted that the severity of growth restriction strongly correlates with oxidative stress markers. In the present study, a correlation was also found between LPO activity in the umbilical cord and newborn length.

In the present study, we found that the SOD activity in the placenta was higher in pregnant women with multiple pregnancies who smoke. Similarly, Pizent et al. [103] found higher SOD activity in the placentas of smoking women compared to non-smokers, while the GPx activity was similar in both groups. Ermis et al. [104] found an increase in GPx activity in the serum of smoking women and their infants. Napierała et al. [105] demonstrated that the GPx, CAT, and SOD activity levels in the serum and milk of smoking women were higher than in non-smokers and those passively exposed to tobacco smoke.

### 4.7. Limitations

In this study, the participants represented only a small proportion of pregnant women in northwestern Poland. Measurements of key iron transport proteins, including transferrin receptor (TfR) and ferroportin (FPN), were not performed. Additionally, hepcidin, a hormone responsible for iron transport to the placenta, was not measured. Notably, hepcidin has been linked to inflammation, which could potentially lead to iron deficiency anemia (IDA). Furthermore, two trans-membrane copper proteins, high-affinity copper uptake protein 1 (CTR1) and DMT1, and zinc transporters Zrt-/Irt-like protein (ZIP) zinc transporters and ZnTs (zinc transporters) were not determined in this study. The levels of albumin and alpha-2-macroglobulin, which commonly form complexes with zinc, were not analyzed. Metallothioneins (MT), which play a vital role in cellular zinc transport, were also not determined.

Additionally, comparable data from twin gestation were not available in the existing scientific literature. Therefore, we included articles reporting umbilical cord blood measurements of Fe, Cu, and Zn. External factors such as diet, environmental pollutants, and the quality of life of pregnant women were not analyzed in this study. It is important for future research to consider the effects of hypoxia on the body’s antioxidant balance. During pregnancy, hypoxia can have negative consequences on fetal development and increase the risk of metabolic and cardiovascular complications. Specifically, hypoxia can lead to the formation of ROS by influencing the mitochondria of the placenta and uterus, which can cause oxidative stress [106,107]. Moreover, we did not consider the placental weight, position, perfusion, and function (determined by Doppler and biochemical tests such as PLGF), which could have a significant impact on fetal development.

However, a strength of this study is its focus on the concentrations of Fe, Cu, and Zn in the placentas of women with twin pregnancies, where specific research is lacking. Additionally, we compared these elements with the morphometric parameters of newborns. The measurement of selected oxidative stress parameters also adds unique significance to the study and provides valuable information about the occurrence of oxidative stress in multiple pregnancies.

## 5. Conclusions

The present study contributes novel information regarding the concentrations of iron, zinc, and copper, as well as the concentrations of lipid peroxidation products and the activities of antioxidant enzymes (superoxide dismutase, catalase, glutathione peroxidase, and glutathione reductase) in the placenta, umbilical cord, and fetal membrane of women with multiple pregnancies. These data can be utilized as comparative data in future studies, although our results should be interpreted with caution despite statistical significance being achieved.

## Figures and Tables

**Table 1:** Maternal and neonatal characteristics (AM, arithmetic mean; SD, standard deviation; Med, median; n, number of participants; BMI, body mass index).

Parameter	AM ± SD	Med	Range
Maternal characteristics:			
Age (years)	31 ± 5	32	21–41
Weight (kg) before pregnancy	64 ± 9	63	53–90
BMI before pregnancy	23 ± 4	24	18–31
Weight (kg) before delivery	84 ± 18	81	81.5–63.0
Weight gain during pregnancy (kg)	18 ± 8	15	8–37
Neonatal characteristics:			
Gestational age(weeks)	35 ± 3	35	26–38
Birthweight (g)	2247 ± 497	2330	690–3350
Length (cm)	48 ± 4	49	38–55
Head circumference (cm)	32 ± 2	32	28–37
Shoulder width (cm)	30 ± 3	30	23–36
Placenta weight (g)	550 ± 231	500	510–1300

**Table 2:** Smoothed centiles for birthweight and birth length of the boys (*n* = 54) and girls (*n* = 65) (Fenton Growth Chart).

Centiles for Length (cm)	Boys	Girls	Total	Centiles for Birthweight (kg)	Boys	Girls	Total
>3 or <97	19	15	34	<3 or <97	21	21	42
<3 or >97	3	8	11	<3 or >97	1	2	3

**Table 3:** An analysis of the reference material Bovine Muscle NIST-SRM 8414.

Element	Reference Values (mg/L)	Percentage of Reference Values (%)
Fe	71.2 ± 9.2	75.8
Zn	142 ± 14	148
Cu	2.84 ± 0.45	3.12

**Table 4:** Concentrations of iron (Fe), zinc (Zn), and copper (Cu) in the placenta, umbilical cord, and fetal membrane in groups for oxidative stress (mg/kg^−1^; dry mass, dw) (AM, arithmetic mean; Med, median; Max, maximum; Min, minimum; SD, standard deviation).

	Placenta			Umbilical Cord			Fetal Membrane		
	AM ± SD	Med	Range	AM±SD	Med	Range	AM ± SD	Med	Range
Total = 21 twins + 1 triplets									
Fe	418 ± 133	412.93	189.19–804.55	200 ± 139	154.27	49.77–640.81	317 ± 117	306.46	80.53–663.75
Zn	50 ± 8	48.98	34.42–68.47	46 ± 78	45.44	32.28–66.08	37 ± 9	35.42	23.78–63.95
Cu	4 ± 1	4.58	1.97–6.2	3 ± 1	3.28	0.85–6.40	7 ± 3	7.31	2.85–13.41

**Table 6:** Spearman’s coefficients between Fe, Zn, and Cu concentrations in the placenta, umbilical cord, and fetal membrane and balance of pro-oxidant/antioxidant and anthropometric parameters of the infants.

	Placenta GPx	Placenta GR	Fetal Membrane CAT	Placenta CAT	Fetal Membrane LPO	Placenta LPO	Umbilical Cord LPO	Placenta SOD	Umbilical Cord SOD
Placenta Zn						0.43 **		0.48 ***	
Umbilical cord Zn								0.48 ***	
Fetal membrane Cu			0.39 *						
Placenta Cu								0.42 **	
Umbilical cord Cu			0.45 ***	0.44 *				0.59 ***	0.50 ***
Fetal membrane Fe					−0.50 ***				
Placenta Fe	0.36 *	0.33 *				0.58 ***			
Length							0.39 *		0.41 **
Head circumference			0.37 *					0.33 *	
Birth weight			0.37 *					0.33 *	0.45 ***
Shoulder width									0.35 *
BMI before pregnancy		0.40 *							

Note: *** *p* <0.001; ** *p* <0.01, * *p* < 0.05.

## Data Availability

The data presented in this study are available on request from the corresponding author.
